# Intermittent fasting inhibits the development of colorectal cancer in APC^*Min*/+^ mice through gut microbiota and its related metabolites

**DOI:** 10.3389/fmicb.2025.1563224

**Published:** 2025-05-29

**Authors:** Jia Chen, Rishun Su, Yulong He, Jiong Chen

**Affiliations:** ^1^Digestive Diseases Center, The Seventh Affiliated Hospital of Sun Yat-sen University, School of Medicine, Sun Yat-sen University, Shenzhen, Guangdong, China; ^2^Department of Anesthesiology, The Seventh Affiliated Hospital of Sun Yat-sen University, School of Medicine, Sun Yat-sen University, Shenzhen, Guangdong, China

**Keywords:** colorectal cancer, intermittent fasting, gut microbiome, isovaleric acid, APC*^Min/+^* mice

## Abstract

**Background and objectives:**

Intermittent fasting is an emerging dietary approach, but its specific role in colorectal cancer (CRC) is not yet clear. In this study, we investigated the relationship between intermittent fasting and colorectal development in mice.

**Methods:**

First, APC^*Min*/+^ mouse models (a spontaneous model of colorectal cancer) were subjected to intermittent fasting intervention (2 days/week) with regular monitoring of body weight changes. Subsequently, 16S rRNA sequencing and untargeted metabolomics were employed to analyze alterations in fecal microbial community structure and metabolic profiles following the fasting intervention. Tumor development was quantitatively assessed by enumerating CRC lesions using HE staining, while histopathological evaluation was performed to determine the degree of neoplastic progression. Concurrently, western blotting was conducted to examine the expression levels of intestinal barrier function-related proteins. Finally, *in vitro* validation experiments, including colony formation assay and transwell invasion assay, were performed to investigate the effects of the key microbial metabolite isovaleric acid on the proliferative and invasive capacities of CRC cells.

**Results:**

Intermittent fasting significantly reduced tumor incidence by approximately 50% compared to the control group (1.25 ± 0.38 vs 2.50 ± 0.38 tumors/mouse, *P* = 0.017) and markedly attenuated tumor progression. 16S rRNA sequencing analysis revealed significant enrichment of two key bacterial genera, *Alistipes* (P = 0.030) and *Odoribacter* (*P* = 0.030), along with a significant reduction in fecal isovaleric acid levels (*P* < 0.05) in the intermittent fasting group. Furthermore, intermittent fasting effectively controlled body weight gain (*P* < 0.05) and significantly improved intestinal barrier function (*P* < 0.05). *In vitro* experiments further demonstrated that isovaleric acid directly promoted CRC cell proliferation (*P* < 0.05) and enhanced their invasive capacity (*P* < 0.05).

**Conclusion:**

Intermittent fasting suppresses CRC development in mice through its effects on gut microbiota and related metabolites.

## 1 Introduction

Colorectal cancer (CRC) is one of the most prevalent and deadly malignancies (Siegel et al., 2024). It causes a significant loss of life and imposes a heavy financial burden each year (Morgan et al., 2040), highlighting the urgent need to explore effective prevention and treatment strategies. CRC is a type of gastrointestinal cancer, and changes in dietary patterns can directly influence its occurrence and progression (Sedlak et al., 2023; Wong and Yu, 2023).

Since ancient Greek times, fasting has been regarded as a beneficial health practice (Yang H. et al., 2024). Modern scientific research suggests that low-calorie diets show surprising effects in cancer prevention and treatment, both in experimental animals and human populations. However, since most people find it difficult to maintain a low-calorie diet over the long term, there is a need for more feasible alternatives. Intermittent fasting (IF) is a dietary approach that is gaining popularity. According to a 2020 survey, intermittent fasting is the most common dietary strategy among adults in the United States.

Previous studies have shown that intermittent fasting has a certain effect on the prevention and treatment of various cancers such as breast, lung, and liver cancer (Clifton et al., 2021; Gallage et al., 2024; Tang et al., 2021; Becker et al., 2023). The study by Gallage et al. (2024) demonstrated that the 5:2 intermittent fasting regimen, involving two non-consecutive fasting days per week, significantly alleviates high-fat diet–induced non-alcoholic fatty liver disease (NAFLD) and suppresses its progression to hepatocellular carcinoma (HCC). The study by Tang et al. (2021) demonstrated that intermittent fasting, in combination with ERK pathway inhibition, enhances the anti-tumor efficacy of chemotherapy against breast and lung cancers by activating the GSK3β–SIRT7 signaling axis. The study by Becker et al. (2023) demonstrated that intermittent fasting enhances the delivery efficiency and antitumor efficacy of nanomedicine in hepatocellular carcinoma by remodeling the tumor microenvironment. However, the precise role of IF in CRC prevention remains insufficiently elucidated, particularly in the context of its interactions with gut microbiota.

Unlike tumors in other parts of the body, CRC is in direct contact with the gut microbiota and interacts with it (Yang J. et al., 2022). The gut microbiota consists of approximately 10^14^ microorganisms, with a gene pool that is 150 times the total number of human genes, and it produces a wide variety of metabolites (Krautkramer et al., 2021; Li et al., 2024). The gut microbiota-metabolite axis is considered a major mechanism through which the gut microbiota participates in the development of CRC (Krautkramer et al., 2021). Microorganisms residing in the gut can metabolize cellulose, which humans cannot directly digest, to produce short-chain fatty acids, thereby participating in the regulation of CRC development and progression (Qu et al., 2023).

Isovaleric acid is a short-chain fatty acid produced by the metabolism of gut microbiota. The role of isovaleric acid in colorectal cancer remains controversial in previous studies, with some studies suggesting a tumor-promoting effect while others propose protective mechanisms (Zhu et al., 2022; Cai et al., 2024).

Building upon these gaps, the present study aimed to investigate whether intermittent fasting could suppress CRC development through modulation of gut microbiota and its metabolites. Using the APC^*Min*/+^ mouse model, we demonstrated that intermittent fasting plays a significant role in inhibiting CRC development. We then elucidated how the changes in the gut microbiota and related derivatives associated with intermittent fasting contribute to the suppression of CRC development. We found that intermittent fasting inhibits CRC development by inducing an increase in beneficial bacteria abundance and suppressing harmful metabolites. Intermittent fasting also protects the intestinal epithelial barrier function.

## 2 Materials and methods

### 2.1 Animal experimental design

APC^*Min*/+^ mice (specific pathogen-free, male, 6 weeks old) were purchased from Cavens Lab Animal Co., Ltd. After a 7 days acclimation period in a controlled environment with constant temperature and humidity, the mice were randomly divided into two groups: the intermittent fasting group and the control group. During the experimental period, mice in the intermittent fasting group underwent fasting twice a week (on non-consecutive days), with each fasting period lasting 24 h. In contrast, the control group had *ad libitum* access to food. During non-fasting periods, the mice were allowed free access to food. The total duration of the experiment was 4 weeks, and all mice had free access to water throughout the study. This fasting regimen was selected based on multiple considerations: it offers high feasibility, is more likely to be accepted and sustained over the long term, and previous studies have demonstrated its potential in cancer prevention as well as in enhancing the efficacy of cancer therapies (Tang et al., 2021; Gallage et al., 2024). At the end of the experiment, fecal samples from both the experimental and control groups were collected for 16S rRNA sequencing and non-targeted metabolomics analysis. At 11 weeks of age, the mice were anesthetized by intraperitoneal injection of pentobarbital sodium and euthanized by cervical dislocation. The intestinal tissues were dissected for further analysis.

### 2.2 Pathological observation of intestinal tissues

The mouse intestinal tissues were fixed in a general-purpose tissue fixative, followed by dehydration with ethanol and paraffin embedding. Paraffin sections were cut at a thickness of 2 μm. Two experienced pathologists independently evaluated the Ileal tissue sections in a double-blind manner. Morphological features were examined under a light microscope at 20 × magnification. The number of tumors in each group was recorded, and pathological grading was performed (*n* = 4 per group).

### 2.3 16S rRNA sequencing of mouse fecal samples

Total DNA from fecal samples of the intermittent fasting group and the control group was extracted using the E.Z.N.A.^<reg>(</reg>^ Soil DNA Kit (Omega Bio-tek, Norcross, GA, United States). The integrity of the extracted DNA was assessed by 1% agarose gel electrophoresis, and DNA concentration and purity were measured using a NanoDrop2000 (Thermo Scientific, United States). The extracted DNA was used as a template for PCR amplification with the upstream primer 338F (5′-ACTCCTACGGGAGGCAGCAG-3′) and the downstream primer 806R (5′-GGACTACHVGGGTWTCTAAT-3′) carrying a barcode sequence. PCR amplification was performed on the 16S rRNA gene V3-V4 variable region. After mixing the PCR products from the same sample, the PCR products were recovered using a 2% agarose gel and purified. The fragment size was assessed by 2% agarose gel electrophoresis, and the recovered products were quantified using a Synergy HTX (Biotek, United States). The purified PCR products were library-prepared using the NEXTFLEX Rapid DNA-Seq Kit (Bioo Scientific, United States). Sequencing was carried out on the Illumina NextSeq 2000 PE300 platform.

### 2.4 High-throughput sequencing data analysis

The raw paired-end sequencing reads were quality controlled using fastp (version 0.19.6)^1^. The paired-end sequences were then merged using FLASH (version 1.2.11)^2^. Sequences were assigned to their respective samples and their starting positions were determined using the barcode and primer information at the beginning and end of the sequences. The direction of the sequences was adjusted based on the barcode and primer information. Denoising was performed using the Qiime2 pipeline to obtain ASVs (Amplicon Sequence Variants). The sequencing depth of all samples was normalized to 20,000 sequences. Taxonomic classification of the ASVs was performed based on the Sliva 16S rRNA gene database (v 138). Functional prediction analysis of 16S was conducted using PICRUSt2 (version 2.2.0).

### 2.5 LC-MS/MS analysis

The LC-MS/MS analysis was efficiently performed using a Thermo UHPLC-Q Exactive HF-X system, with technical support provided by Majorbio Bio-Pharm Technology Co., Ltd. (Shanghai, China).

A 3 μL sample was separated using an HSS T3 chromatographic column (100 mm × 2.1 mm i.d., 1.8 μm) and analyzed by mass spectrometry. Mobile phase A consisted of 95% water and 5% acetonitrile (with 0.1% formic acid), and mobile phase B consisted of 47.5% acetonitrile, 47.5% isopropanol, and 5% water (with 0.1% formic acid). The flow rate was 0.40 mL/min, and the column temperature was set to 40°C.

Mass spectrometric detection was performed in both positive and negative ion modes. The mass scan range was from 70 to 1,050 m/z. The sheath gas flow rate was set to 50 psi, and the auxiliary gas flow rate was 13 psi, with an auxiliary gas heater temperature of 425°C. The ion spray voltage was 3,500 V in positive mode and −3,500 V in negative mode. The ion transfer tube temperature was set to 325°C. The normalized collision energy was 20-40-60 V in a cyclic collision energy mode. The first-stage MS resolution was 60,000, and the second-stage MS resolution was 7,500. Data were acquired in DDA mode.

### 2.6 Metabolite identification and analysis

The raw LC-MS data were processed using Progenesis QI software, which included peak identification, retention time correction, and other preprocessing steps. Metabolite identification was carried out by matching the detected features against the HMDB, Metlin, and in-house databases. After data processing, which involved applying the 80% rule for missing values, sum normalization, and QC filtering (with RSD < 30%), multivariate statistical analysis was performed using the ropls package. To identify significantly differential metabolites, criteria of VIP > 1 and *p* < 0.05 were applied, and orthogonal partial least squares discriminant analysis (OPLS-DA) was employed.

### 2.7 Western blot analysis

Total protein from intestinal tissues was extracted using RIPA lysis buffer (Beyotime, Beijing, China) and quantified with a BCA protein assay kit (KeyGEN, Jiangsu, China). Equal amounts of protein (20 μg) were separated by 10% SDS-PAGE (Bio-Rad system) and transferred onto 0.45 μm PVDF membranes (Millipore). The membranes were blocked with 5% non-fat milk at room temperature for 1 h and then incubated overnight at 4°C with primary antibodies against ZO-1 (1:1000), E-cadherin (1:1000), PCNA (1:1000), and GAPDH (1:500) (EpiZyme, Shanghai, China). After washing with TBST, membranes were incubated with HRP-conjugated secondary antibody (1:2000, Beyotime, Beijing, China) at room temperature for 1 h. Protein bands were visualized using an enhanced chemiluminescence (ECL) detection reagent (Meilunbio, Dalian, China). Band intensities were quantified using ImageJ software, and target protein expression levels were normalized to GAPDH.

### 2.8 Data statistics

All data are presented as mean ± standard deviation (mean ± SD). Each group consisted of four mice. Intergroup comparisons were performed using appropriate statistical tests based on data characteristics: the unpaired Student’s *t*-test for variables assumed to follow normal distribution (e.g., intestinal tumor counts) and the Wilcoxon rank-sum test for variables with potential non-normal distributions (e.g., α-diversity indices). For categorical variables between two groups, the chi-square test (χ^2^ test) or Fisher’s exact test was used. All statistical analyses were conducted using GraphPad Prism (version 9.0, GraphPad Software, La Jolla, CA, United States). A *P*-value < 0.05 was considered statistically significant.

## 3 Results

### 3.1 Effect of intermittent fasting on the body weight of APC^Min/+^ mice

**FIGURE 1:**
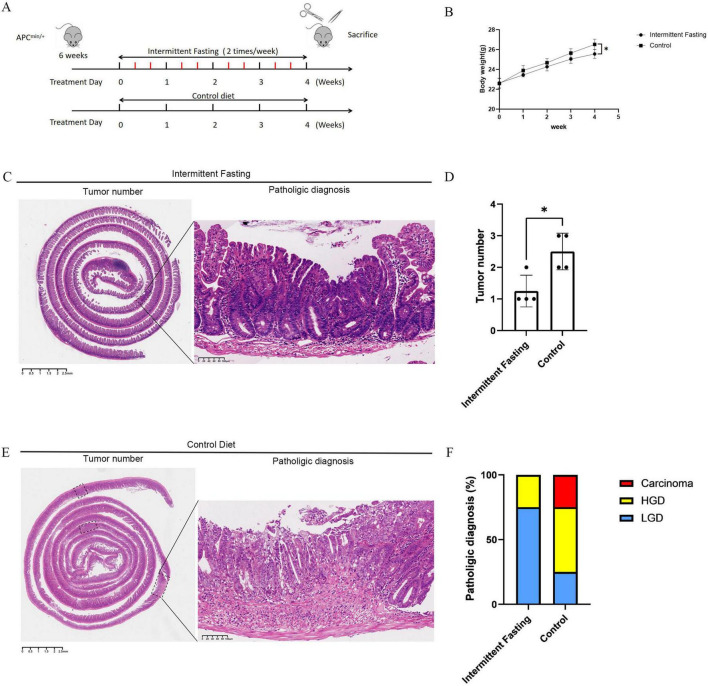
Intermittent fasting inhibits colorectal cancer (CRC) development in APC^Min/+^ mice. **(A)** Animal experiment schematic diagram; **(B)** Body weight change graph of mice during the experiment; **(C)** Representative image of CRC in mice with intermittent fasting; **(D)** Statistical chart of CRC taxonomy in mice of the intermittent fasting group and the control group; **(E)** Representative image of CRC in mice of the control group; **(F)** Statistical chart of the degree of CRC lesions in mice of the intermittent fasting group and the control group. *Indicates *p* < 0.05.

To investigate the inhibitory effect of intermittent fasting on CRC development, the intermittent fasting mice underwent fasting for two non-consecutive days per week for a total of 4 weeks, while the control group mice were allowed *ad libitum* access to food throughout the experimental period (Figure 1A). During the modeling process, the mice in the intermittent fasting group exhibited healthy appearances, with shiny fur and no signs of blood in the stool or rectal prolapse, whereas one mouse in the control group developed rectal prolapse and blood in the stool. There was no significant difference in body weight between the two groups at the start of the experiment. However, from the first week of the experiment, the body weight of the mice in the intermittent fasting group was slightly lower than that of the control group. As the experiment progressed, this trend became more pronounced in the second and third weeks. By the fourth week, the body weight of the intermittent fasting group mice was significantly lower than that of the control group (25.56 ± 0.32 vs 26.52 ± 0.32 g, *P* = 0.0235) (Figure 1B).

### 3.2 Intermittent fasting inhibits tumor development in APC^Min/+^ mice

Hematoxylin and eosin (HE) staining results showed that the number of intestinal tumors in the intermittent fasting group was significantly lower than that in the control group (1.25 ± 0.38 vs 2.50 ± 0.38 g, *P* = 0.0170) (Figures 1C–E). Pathological diagnoses from HE staining also showed that the incidence of intestinal tumor malignancy in the intermittent fasting group was significantly lower than that in the control group (Figure 1F).

### 3.3 Intermittent fasting inhibits intestinal cell proliferation and improves intestinal barrier function in APC^Min/+^ mice

**FIGURE 2:**
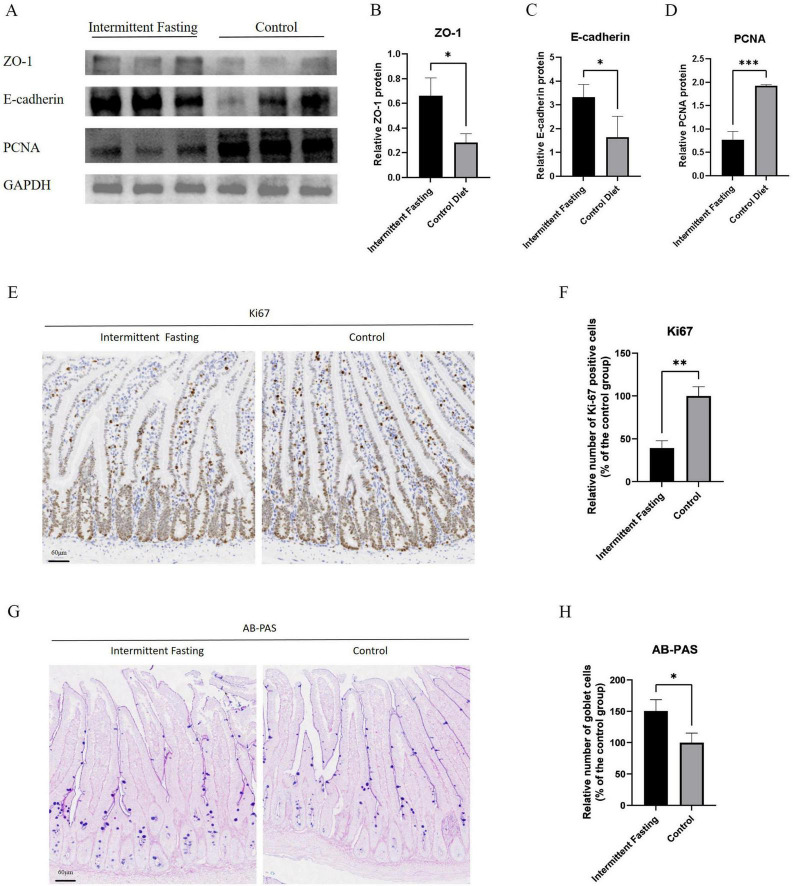
Intermittent fasting reduces intestinal cell proliferation and enhances intestinal barrier function in APC^Min/+^ mice. **(A)** Western blot analysis of ZO-1, E-cadherin, and PCNA in APC^Min/+^ mice of the intermittent fasting group and the control group; **(B)** Statistical analysis of Western blot results for ZO-1 in APC^Min/+^ mice of the intermittent fasting group and the control group; **(C)** Statistical analysis of Western blot results for E-cadherin in APC^Min/+^ mice of the intermittent fasting group and the control group; **(D)** Statistical analysis of Western blot results for PCNA in APC^Min/+^ mice of the intermittent fasting group and the control group; **(E)** Representative immunohistochemical images of Ki67 in APC^Min/+^ mice of the intermittent fasting group and the control group; **(F)** Statistical chart of Ki67 immunohistochemistry in APC^Min/+^ mice of the intermittent fasting group and the control group; **(G)** Representative AB-PAS images of APC^Min/+^ mice in the intermittent fasting group and the control group; **(H)** Statistical chart of AB-PAS staining in APC^Min/+^ mice of the intermittent fasting group and the control group. *, **, and ***indicate *p* < 0.05, *p* < 0.01, and *p* < 0.001, respectively.

To investigate the effect of intermittent fasting on the intestinal barrier function in APC^Min/+^ mice, we performed immunoblotting to measure the expression levels of intestinal barrier markers ZO-1 and E-cadherin in the intestinal tissues of both the intermittent fasting and control groups. The results showed that the expression levels of ZO-1 (*P* < 0.05) and E-cadherin (*P* < 0.05) were significantly higher in the intermittent fasting group compared to the control group, indicating enhanced intestinal barrier function in the intermittent fasting group (Figures 2A–C). Regarding cell proliferation capacity, both PCNA immunoblotting (*P* < 0.001) and Ki67 (*P* < 0.01) immunohistochemistry results indicated a reduced proliferative capacity of intestinal cells in the intermittent fasting group (Figures 2A, D–F).

Furthermore, AB-PAS staining revealed a significant increase in the number of goblet cells in the intermittent fasting group compared to the control group (*P* < 0.05) (Figures 2G, H), further supporting the notion that intermittent fasting enhances intestinal barrier function.

### 3.4 Intermittent fasting alters gut microbiota composition in APC^Min/+^ mice

**FIGURE 3:**
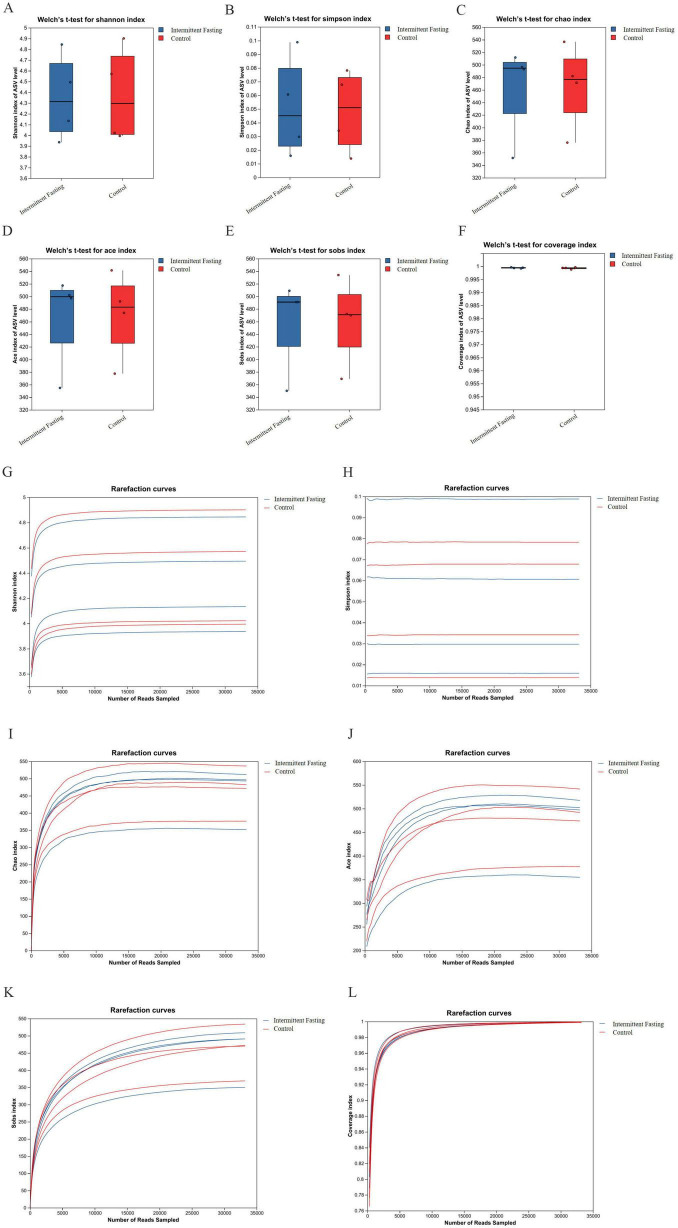
Box plot of intestinal microbiota alpha diversity and Rarefaction curve in APC^Min/+^ mice of the intermittent fasting group and the control group. **(A–F)** Shannon/ Simpson/ Chao/ Ace/ Sobs/ Coverage index box plot of APC^Min/+^ mice in the intermittent fasting group and the control group; **(G–L)** Rarefaction curve of Shannon/ Simpson/ Chao/ Ace/ Sobs/ Coverage index in APC^Min/+^ mice of the intermittent fasting group and the control group.

**FIGURE 4:**
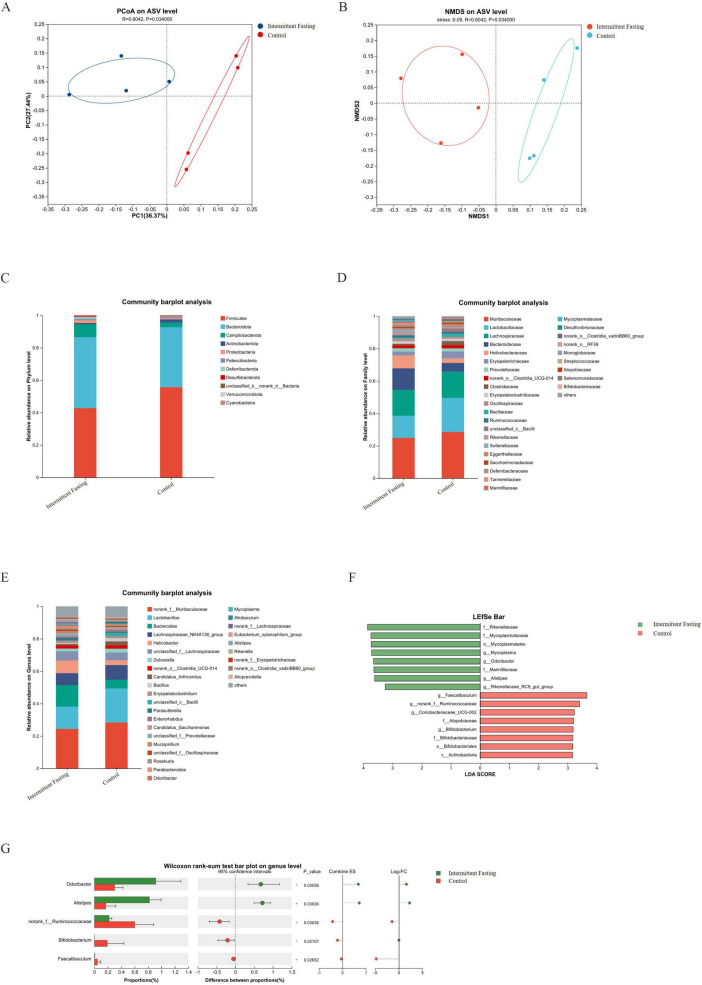
Intermittent fasting significantly alters the gut microbiota composition in APC^Min/+^ mice. **(A,B)** PcoA/ NMDS analysis plot of intestinal microbiota in APC^Min/+^ mice of the intermittent fasting group and the control group; **(C–E)** Species composition of intestinal microbiota at the phylum/ class/ genus levels in APC^Min/+^ mice of the intermittent fasting group and the control group; **(F)** LEfSe analysis plot of intestinal microbiota in APC^Min/+^ mice of the intermittent fasting group and the control group; **(G)** Differential analysis of intestinal microbiota at the genus level in APC^Min/+^ mice of the intermittent fasting group and the control group.

To explore the role of gut microbiota in the suppression of CRC development related to intermittent fasting, we performed microbiome analysis on fecal samples from intermittent fasting and control mice. The α diversity analysis showed that there was no significant difference in the gut microbiota α diversity between the intermittent fasting group and the control group of mice (*P* > 0.05) (Figures 3A–F). The rarefaction curve of the alpha diversity index showed that as sequencing depth increased, the curve gradually plateaued, indicating that diversity had approached saturation (Figures 3G–L). This suggested that the sequencing depth was sufficient and that further increasing it would not significantly add to the discovery of new species, confirming the reliability of the sequencing data. As for β diversity, principal coordinate analysis confirmed the differences in microbiota composition between the intermittent fasting and control groups (*P* < 0.05) (Figures 4A, B). Community composition analysis at the phylum level showed that, regardless of the group, the most abundant phyla were *Firmicutes* and *Bacteroidetes*. However, we observed that the percentage of *Firmicutes* was lower in the intermittent fasting group compared to the control group, while the level of *Bacteroidetes* was elevated (Figure 4C). At the family level, *Bacteroidaceae* and *Helicobacteraceae* were increased in the intermittent fasting group, whereas *Muribaculaceae* and *Lactobacillaceae* were decreased compared to the control group (Figure 4D). At the genus level, *norank_f_Muribaculaceae* and *Lactobacillus* were lower in the intermittent fasting group, while *Bacteroides* was higher (Figure 4E). *Odoribacter* (*P* < 0.05) and *Alistipes* (*P* < 0.05) were significantly elevated in the intermittent fasting group compared to the control group, while *norank_f_Ruminococcaceae* (*P* < 0.05), *Bifidobacterium* (*P* < 0.05), and *Faecalibaculum* (*P* < 0.05) were significantly decreased (Figure 4F). LEfSe analysis showed that *Odoribacter* and *Alistipes* were among the most significantly altered genera in the intermittent fasting group (Figure 4G).

### 3.5 Intermittent fasting alters gut metabolism in APC^Min/+^ mice

**FIGURE 5:**
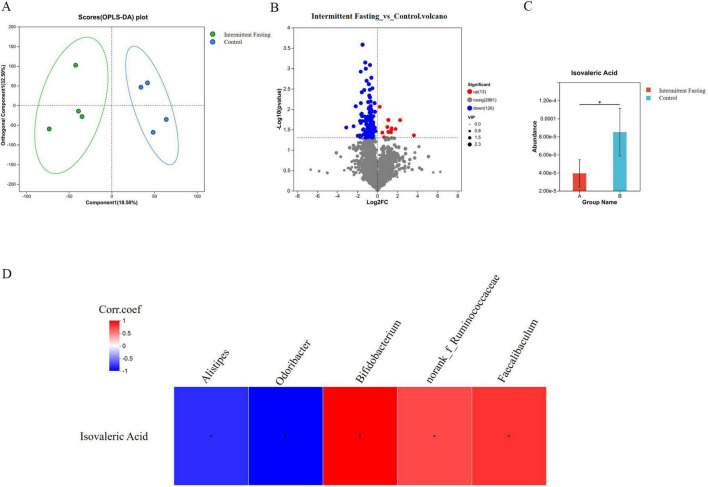
Intermittent fasting alters the gut metabolite composition in APC^Min/+^ mice. **(A)** OPLS-DA analysis of gut metabolites in APC^Min/+^ mice of the intermittent fasting group and the control group; **(B)** Differential analysis of gut metabolites in APC^Min/+^ mice of the intermittent fasting group and the control group; **(C)** Differential analysis of isovaleric acid in the gut of APC^Min/+^ mice in the intermittent fasting group and the control group; **(D)** Correlation analysis plot between isovaleric acid and differential gut microbiota genera. *Indicates *p* < 0.05.

To reveal the metabolic changes induced by intermittent fasting, we performed metabolic analysis on the fecal samples from APC^Min/+^ mice. OPLS-DA showed that the dots representing the intermittent fasting and control groups clustered within each group and were separated between groups (Figure 5A). Differential metabolites were identified in the intermittent fasting mice (Figure 5B), including isovaleric acid, a common short-chain fatty acid metabolite of the gut microbiota. Differential analysis indicated that isovaleric acid levels were significantly (*P* < 0.05) reduced in the intermittent fasting group compared to the control group, with the fasting group displaying approximately 46% of the isovaleric acid levels observed in the control group (Figure 5C).

We conducted a comprehensive analysis to determine the potential association between gut microbiota changes and metabolites in the intermittent fasting mice. We found that *Alistipes* (*P* < 0.05) and *Odoribacter* (*P* < 0.01) were significantly negatively correlated with isovaleric acid, while *Bifidobacterium* (*P* < 0.01), *norank_f_Ruminococcaceae* (*P* < 0.05), and *Faecalibaculum* (*P* < 0.05) were significantly positively correlated with isovaleric acid (Figure 5D). These results suggest that the altered gut microbiota and its associated metabolites may contribute to the suppression of colorectal tumorigenesis related to intermittent fasting.

### 3.6 Metabolites altered by intermittent fasting contribute to cell proliferation and metastasis

**FIGURE 6:**
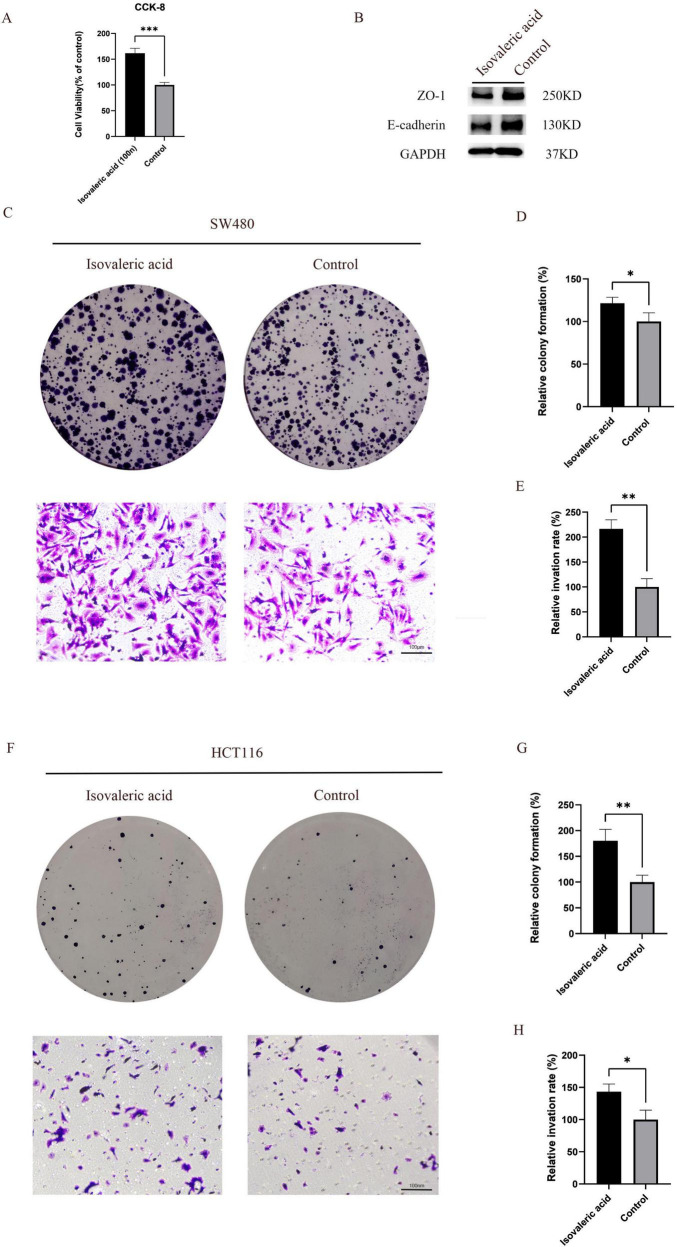
Isovaleric acid promotes the proliferation and migration of colorectal cancer (CRC) cells. **(A)** Isovaleric acid promotes the viability of CRC cells; **(B)** Isovaleric acid disrupts the intestinal barrier function; **(C–E)** Isovaleric acid inhibits the proliferation and migration ability of SW480 CRC cells; **(F–H)** Isovaleric acid inhibits the proliferation and migration ability of HCT116 CRC cells. *, **, and ***indicate *p* < 0.05, *p* < 0.01, and *p* < 0.001, respectively.

To investigate the potential functional role of metabolites altered by intermittent fasting in CRC progression, we conducted *in vitro* experiments. CCK8 assays demonstrated that isovaleric acid enhanced the proliferation of CRC cells (*P* < 0.001) (Figure 6A). Furthermore, Western blot analysis revealed that E-cadherin and ZO-1 protein expression levels were reduced in CRC cells treated with isovaleric acid, suggesting a potential impairment of intestinal barrier function (Figure 6B).

To further explore its effects, we treated two CRC cell lines (SW480 and HCT116) with isovaleric acid. The results showed that isovaleric acid significantly promoted the proliferation of SW480 (*P* < 0.05) and HCT116 (*P* < 0.01) cells (Figures 6C, D, F, G). Additionally, Transwell invasion assays indicated that isovaleric acid enhanced the invasive capacity of SW480 (*P* < 0.01) and HCT116 (*P* < 0.05) cells (Figures 6C, E, F, H).

**FIGURE 7:**
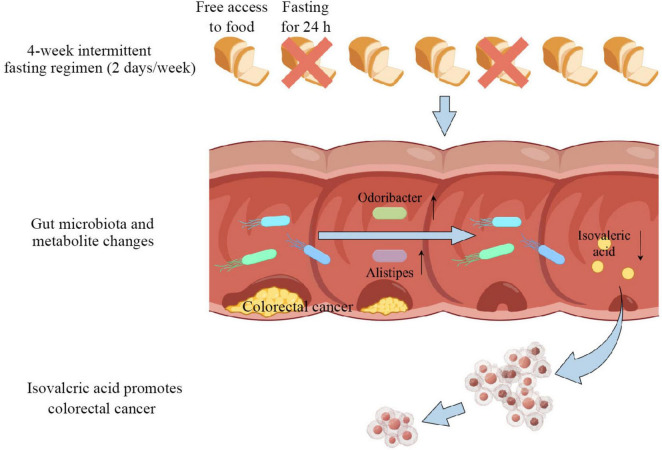
Mechanism diagram of intermittent fasting inhibiting the development of colorectal cancer (CRC).

In summary, these results suggest that intermittent fasting, at least in part, inhibits CRC development by suppressing the carcinogenic metabolite isovaleric acid (Figure 7).

## 4 Discussion

In this study, we demonstrated that intermittent fasting effectively suppresses colorectal tumor development in APC^Min/+^ mice. The gut microbiota and its associated metabolite, isovaleric acid, play a critical role in this process. Intermittent fasting markedly altered the composition of the gut microbiota, leading to an increased abundance of genera such as *Odoribacter* and *Alistipes*. These changes may contribute to a reduction in isovaleric acid levels by reshaping the intestinal metabolic environment or through antagonistic interactions with isovaleric acid-producing microbes. Previous studies have shown that isovaleric acid can activate the Wnt/β-catenin signaling pathway, a key driver of tumorigenesis in APC^Min/+^ mice (Lähde et al., 2021; Zhu et al., 2022). Therefore, our findings suggest that intermittent fasting may exert its antitumor effects by modulating the gut microbiota and its metabolites, thereby indirectly inhibiting the activation of oncogenic signaling pathways.

We confirmed that intermittent fasting significantly reduced colorectal development in the APC^Min/+^ mouse model. This finding is supported by previous research, which has shown that fasting can suppress cell proliferation and delay tumor progression (Weng et al., 2020; Luo et al., 2024). Intermittent fasting typically leads to weight loss, which has been shown to be negatively correlated with the risk of CRC (Mandic et al., 2023). This is consistent with our findings, as the mice in the intermittent fasting group had lower body weight compared to the control group.

Multiple studies have shown that the composition of the gut microbiota is primarily influenced by diet (Song et al., 2020; Cornish et al., 2024; Ross et al., 2024), and that changes in the gut microbiota are involved in the entire process of CRC initiation and progression (Li et al., 2023; Wang et al., 2024; Yang H. et al., 2024). To investigate the impact of intermittent fasting on the gut microbiota of mice, we first performed 16S rRNA sequencing on the fecal samples from the intermittent fasting and control groups, followed by bioinformatics analysis of the high-throughput data. Beta diversity analysis revealed significant differences in the gut microbiota between the intermittent fasting and control groups. This result is consistent with previous population-based clinical studies and animal-based experimental data. Su et al.’s (2021) research demonstrated that Ramadan-associated intermittent fasting alters the gut microbiota structure. Francesca Cignarella et al.’s (2018) study demonstrated that intermittent fasting mice exhibited significant changes in gut microbiota structure compared to the control group. To further investigate the impact of intermittent fasting on the gut microbiota, we analyzed microbial composition across different taxonomic levels. Notably, the *Firmicutes/Bacteroidota* (F/B) ratio was reduced in the intermittent fasting group. Previous studies have shown that the F/B ratio is closely associated with obesity, with obese individuals typically exhibiting an elevated F/B ratio. In a clinical study, obese patients undergoing multidisciplinary collaborative weight management (MCWM)—which included energy-restricted dietary intervention—showed reductions in both body weight and the F/B ratio (Zhao et al., 2025). These findings are consistent with the concurrent decreases in body weight and F/B ratio observed in the intermittent fasting group in our study, suggesting that changes in the F/B ratio may reflect the dual modulatory effects of dietary interventions on host metabolic status and gut microbial ecolo. At the genus level, intermittent fasting led to a significant enrichment of *Odoribacter* and *Alistipes*. These genera have been identified in previous high-quality studies as anti-inflammatory microbes capable of producing short-chain fatty acids (SCFAs), which contribute to the alleviation of intestinal inflammation—a well-established risk factor for CRC (Guo et al., 2021; Meng et al., 2024). LEfSe analysis further validated *Odoribacter* and *Alistipes* as key microbial signatures associated with the intermittent fasting-induced shift in gut microbiota composition. Collectively, these findings suggest that the increased abundance of *Odoribacter* and *Alistipes* may contribute to the inhibitory effects of intermittent fasting on CRC development.

Since the gut microbiome-metabolism axis is one of the main ways through which the gut microbiome regulates host health (Krautkramer et al., 2021), we performed untargeted metabolomics sequencing to detect changes in metabolites in the feces of intermittent fasting mice and control mice. OPLS-DA analysis showed that the gut metabolites of the two groups of mice were clearly separated, although not significantly. In the differential metabolite analysis, we found a significant decrease in the short-chain fatty acid isovaleric acid in the intermittent fasting group. We performed a correlation analysis to investigate the potential association between gut microbiota alterations induced by intermittent fasting and the differential metabolite isovaleric acid. Our results revealed that *Alistipes* and *Odoribacter* were significantly negatively correlated with isovaleric acid, suggesting a possible involvement of these genera in isovaleric acid metabolism. Although causality cannot be established at this stage, previous studies have reported that *Alistipes* and *Odoribacter* primarily produce short-chain fatty acids such as butyrate and acetate, rather than isovaleric acid (Zheng et al., 2021; Meng et al., 2024; Zhang et al., 2024). Therefore, it is plausible that these bacteria may suppress isovaleric acid production indirectly, either by competing for substrates or by modulating the metabolic environment of the gut. Alternatively, they may exhibit antagonistic interactions with isovaleric acid-producing microorganisms. These results suggest that the altered gut microbiota and its associated metabolites may contribute to the inhibition of CRC development associated with intermittent fasting.

The role of isovaleric acid in CRC remains controversial. While some studies suggest that this short-chain fatty acid may have protective effects against CRC (Cai et al., 2024), emerging evidence points to its potential tumor-promoting role. A large-scale cohort study involving 616 participants identified a significant positive correlation between isovaleric acid levels and colorectal cancer progression through metabolomics analysis, with isovaleric acid concentrations showing a progressive increase from early-stage (S0) to advanced-stage (SIII/IV) cancer (Yachida et al., 2019). This clinical finding corroborates the results of Pingping Zhu’s team, which similarly confirmed the enrichment of the gut microbiota metabolite isovaleric acid in colorectal cancer using a DSS/AOM-induced mouse model and patient metabolomics sequencing data. Mechanistically, isovaleric acid upregulates Tph2 in intestinal serotonergic neurons, increasing 5-HT production, which activates the HTR1B/1D/1F-Wnt/β-catenin pathway in cancer stem cells (CSCs) to promote self-renewal and tumorigenesis (Zhu et al., 2022). Given that one study relies on large-scale clinical data and the other integrates both clinical data and *in vivo* experimental validation, these findings provide strong evidence supporting the tumor-promoting role of isovaleric acid in CRC. Additionally, the significantly lower levels of isovaleric acid observed in the intermittent fasting group compared to the control group further suggest that isovaleric acid may contribute to the development of CRC. To verify this, we conducted CCK8, colony formation, and Transwell assays. The results showed that isovaleric acid significantly promoted the growth, colony formation, and invasive ability of CRC cells. Previous studies have shown that isovaleric acid enhances the expression of Wnt/β-catenin target genes (c-Myc, Axin2, Ccnd1, and Lgr5) in colon tissue (Zhu et al., 2022). Therefore, we hypothesize that isovaleric acid may promote CRC development by activating the Wnt/β-catenin pathway in APC^Min/+^ mice.

Although the results of this study indicate that intermittent fasting significantly alters the gut microbiota in mice and support the role of the microbiota-derived metabolite isovaleric acid in promoting CRC progression, several limitations should be considered. First, the study employed the APC^Min/+^ spontaneous CRC mouse model, which warrants caution when extrapolating these findings to clinical settings. Notably, intermittent fasting may induce physiological stress or metabolic disturbances, such as hunger, peptic ulcers, and hypoglycemia, which could confound the results. Second, CRC development is likely influenced by a combination of multiple factors. While a significant correlation between isovaleric acid and CRC progression was observed, it is important not to overstate its direct causative role in tumorigenesis. Additionally, the current data do not completely rule out the possibility of indirect effects.

To further clarify the causal relationships, we plan to conduct fecal microbiota transplantation (FMT) experiments in future studies.

## 5 Conclusion

This study confirms that intermittent fasting inhibits the development of CRC in mice by modulating the gut microbiota and its associated metabolites. These findings provide potential theoretical references for the development of dietary intervention strategies for CRC prevention and offer possible insights for clinical dietary interventions.

## Data Availability

The data presented in the study are deposited in the NCBI repository, accession number PRJNA1260685.

## Footnotes

1
https://github.com/OpenGene/fastp

2
http://www.cbcb.umd.edu/software/flash
